# APTT and D-dimer as biomarkers for heatstroke in patients with severe heat-related illnesses

**DOI:** 10.1097/MD.0000000000039493

**Published:** 2024-08-30

**Authors:** Xu Li, Yuanjie Wang

**Affiliations:** a Department of Blood Transfusion, Suining Central Hospital, Sichuan, Suining, PR China.

**Keywords:** APTT, biomarkers, D-dimer, heatstroke

## Abstract

The objective of this study was to analyze the changes of activated partial thromboplastin time (APTT) and D-dimer in severe heatstroke (HS) patients and their value in identifying HS patients and to analyze clinical features and early laboratory test results of heat-related illnesses. Forty-five patients with heat-related illnesses who were admitted to the Department of Emergency and Intensive Care Medicine of Suining Central Hospital from June 2022 to April 2023 were retrospectively analyzed. Patients were divided into 3 groups based on their clinical diagnosis: classic HS group, exertional HS group, and control group. General date and laboratory test results were collected, especially APTT and D-dimer. The receiver operating characteristic curve was used to analyze D-dimer and APTT. : There were differences in gender distribution among the 3 groups. Exertional HS was dominated by male patients, and classic HS was dominated by elderly patients. Binary logistic regression analysis of coagulation index showed a significant correlation between D-dimer and APTT and HS. The receiver operating characteristic curve results showed that APTT and D-dimer had high sensitivity and specificity in the identification of HS with an area under the curve (AUC) of 0.846, sensitivity of 97%, and specificity of 58.3% for APTT and an AUC of 0.861, sensitivity of 72%, and specificity of 91.7% for D-dimer (D-dimer + APTT [AUC, 0.929; sensitivity, 81.8%–91.7%; *P* < .001]). : The mortality rate of HS is high, and early diagnosis is particularly important. APTT and D-dimer may be used as markers assisting in identifying HS.

## 1. Introduction

The heat-related illnesses include 3 different manifestations, heat cramps, heat exhaustion, and heatstroke (HS), which refers to the imbalance of heat production and heat dissipation in the body in a high temperature environment, resulting in a critical illness characterized by abnormal function of the central nervous system, accompanied by systemic damage. HS is the most severe and has the worst prognosis. It is divided into 2 categories, exertional HS (EHS) and classic HS (CHS), depending on whether there are causes of physical labor in a hot environment. With the global climate change, the incidence of heat disease is increasing, but there is a lack of specific laboratory indicators to especially help in identifying HS. Studies have shown that one of the reasons for its high mortality is the occurrence of abnormal coagulation function and disseminated intravascular coagulation (DIC).^[1]^ Heat-induced vascular endothelial injury, leading to inappropriate aggregation of platelets, may be the initial link of the whole thermal injury–induced coagulation abnormalities. In some animal experiments, the change of D-dimer levels was found to be correlated with the severity of thermal injury and accompanied the changes of organ damage markers. But whether it can affect the prognosis by improving coagulation has not been confirmed. There is no panacea for the treatment of HS, and cooling remains the most important therapeutic relief, but changes in body temperature do not seem to ameliorate inappropriate activation of the coagulation system. With the increase of the incidence of HS, improving the understanding of HS, how to prevent it, and exploring the factors affecting the prognosis are hot topics in recent years. We analyzed the changes of activated partial thromboplastin time (APTT) and D-dimer in HS through a retrospective case-control study, which may provide reference for the clinician and improve the understanding of HS.

## 2. Materials and methods

### 2.1. Patients

Forty-five patients with severe heat-related illnesses who were admitted to the Department of Emergency and Intensive Care Medicine of Suining Central Hospital from May 2022 to May 2023 were retrospectively analyzed. The inclusion criteria were as follows: (1) comply with the diagnostic criteria of the Chinese expert consensus on the diagnosis and treatment of HS disease^[2]^ and (2) aged over 18 years. The exclusion criteria were as follows: (1) patients with original organ failure; (2) those with previous coagulation disorders; (3) those with underlying diseases treated with anticoagulants, for instance, patients with heart disease who require long-term warfarin or those with coronary heart disease who take long-term antiplatelet drugs; and (4) those with any malignancy. Patients with HS were further divided into EHS (n = 13) and CHS (n = 20) based on whether there were labor factors under high temperature conditions as the inducement of the illness. The control group (n = 12) included patients diagnosed with heat cramps and heat exhaustion, which are generally considered milder heat-related illnesses.^[3]^

### 2.2. Clinical data

The general information of patients included gender, age, onset time, body temperature at admission, and heart rate. Laboratory tests included alanine transaminase, serum creatinine, procalcitonin, prothrombin time (PT), APTT, D-dimer, fibrinogen, white blood cell, neutrophil, and platelet. Patients were followed up for 28 days, and the primary end point was 28-day all-cause mortality. The number of survival and death cases was recorded.

### 2.3. Blood test method

All blood sample collection was completed within 4 hours after admission, and all coagulation tests were completed within 8 hours after admission. Venous blood was collected using a 3.8% sodium citrate anticoagulant tube during fasting in both groups. All coagulation parameters were measured by STA Compact, a fully automated coagulation analyzer produced in Germany.

### 2.4. Statistical analysis

All data were statistically processed using SPSS22.0, and Shapiro–Wilk method was selected for normality test. Measurement data conforming to normal distribution were expressed as mean ± standard deviation ($\bar{x}\pm s$). Normal data including age, prehospital time, heart rate, white blood cells, body temperature, PT, APTT, and platelets were compared among multiple groups by 1-way ANOVA. The measurement data of non-normal distribution were expressed by the median and quartile distance (median [P25, P75]). Non-normal data included neutrophil ratio, alanine aminotransferase, creatinine, and procalcitonin. The Mann–Whitney *U* test was used for comparison. Statistical data including sex and mortality were compared by χ^2^ test. logistic regression analysis was performed to analyze the correlation between coagulation indexes and the diagnosis of thermal radiation disease. The receiver operating characteristic (ROC) curve was used to calculate the sensitivity and specificity of APTT and D-dimer to help identify HS, and the best cutoff value of each index was calculated. *P* < .05 was considered statistically significant.

## 3. Results

### 3.1. General situation

Clinical baseline parameters were comparable among the 3 groups (Table 1). The EHS group had more male patients, whereas the CHS group had more elderly patients. The overall average age was 68.27 ± 12.067 years, and patients with CHS were elder than those in the EHS group (76.15 ± 7.44 vs 56.08 ± 9.46 years old; *P* < .05). ESH patients were more likely to suffer from a sever liver damage, with a rate of 61.5% (8/13). Whatever, the control group’s liver conditions were better. Blood culture was performed in 2 cases of control group, 3 cases of CHS group, and 5 cases of EHS group, but the results were all negative.

**Table 1:** General situation and lab examination of patients in 3 groups.

	Control group (n = 12)	CHS (n = 20)	EHS (n = 13)	*F/x^2^/H*	*P* value
Sex (M/F)	5/7	12/8	11/2*	4.78	.029†
Age (yr)	68.33 ± 10.03	76.15 ± 7.44*‡	56.08 ± 9.46*	20.63	<.001†
Time (h)	18.83 ± 21.48	26.65 ± 37.39	37.69 ± 43.68	0.87	.426
Heart	81.75 ± 17.04	102.15 ± 20.21*	96.54 ± 16.70*	4.64	.015†
WBC (10^9^/L)	11.43 ± 5.22	13.6 ± 5.97	13.62 ± 5.46	0.423	.658
T (°C)	38.92 ± 1.00	41.25 ± 1.07*	40.77 ± 0.60*	4.42	<.001†
NE (%)	80.61 (65.59–80.20)	86.68 (80.51–86.68)*	85.43 (75.89–89.51)*	4.31	.02†
ALT (U/L)	21 (17.50–50.50)	38.50 (26.25–111.00)*‡	84.00 (31.00–105.00)*	3.935	.027†
Scr (µmol/L)	65 (52.25–122.5)	125 (82.50–181.25)	134 (117.00–217.50)	2.231	.12
PCT (ng/mL)	6 (3.25–12.25)	17 (5.0–45.75)	5 (5.50–22.50)	1.23	.229
Mortality (n, %)	1 (8.30%)	14 (70.0%)*	8 (61.5%)*	13.72	<.001†
Dizzy	8 (66.67%)	16 (80.0%)	10 (76.92%)	0.35	.706
Emesis	3 (25.0%)	8 (40.0%)	6 (46.15%)	0.60	.55
Shock	0 (0.0%)	6 (20.0%)	2 (15.38%)	1.132	.304

ALT = alanine transaminase, CHS = classic heatstroke, EHS = exertional heatstroke, F = female, M = male, NE = neutrophile, PCT = procalcitonin, Scr = serum creatinine, T = temperature, WBC = white blood cell.

* Compared with CHS *P* < .05.

† *P* < .05.

‡ Compared with EHS *P* < .05.

### 3.2. APTT and D-dimer

The coagulation factors of the 3 groups are compared in Table 2. APTT in the EHS group was significantly longer than that in the control group (*P* < .05), D-dimer was increased in both EHS and CHS groups, especially in the EHS group; platelet in the CHS group was lower than that in the control group, but there was no significant difference between the EHS group and the CHS group.

**Table 2:** Coagulation indicators in 3 groups.

	Control group (n = 12)	CHS (n = 20)	EHS (n = 13)	*t/H*	*P*
PT (s)	14 ± 2.80	18 ± 12.90	33.08 ± 38.93	2.554	.09
APTT (s)	30.58 ± 6.43	48 ± 32.50	55.31 ± 24.97*	3.057	.035†
PLT (10^9^/L)	117.42 ± 52.65	66 ± 70.50*	75.46 ± 46.53	2.529	.039†
FIB (g/L)	2 (2.0–2.75)	2 (2.0–3.0)	1 (1.5–3.0)	0.174	.841
D-dimer (µg/L)	184 (20–421)	617 (301.5–711.0)*‡	758.5 (644.0–934.5)*	8.063	.001†

APTT = activated partial thromboplastin time, CHS = classic heatstroke, EHS = exertional heatstroke, FIB = fibrinogen, PLT = platelet, PT = prothrombin time.

* Compared with CHS *P* < .05.

† *P* < .05.

‡ Compared with EHS *P* < .05.

The results of binary logistic regression for analyzing factors related to HS are presented in Table 3. APTT and D-dimer were significantly correlated with HS with odds ratios of 1.227 and 1.005, respectively (*P* < .05).

**Table 3:** Binary logistic regression of the association between coagulation parameters and heatstroke.

	β	SE	*P* value	OR	95% CI
PT (s)	0.196	0.133	.139	1.217	0.938 ± 1.578
APTT (s)	0.205	0.08	.011*	1.227	1.048 ± 1.436
D-dimer (ng/L)	0.005	0.002	.002*	1.005	1.002 ± 1.008
FIB (g/L)	0.223	0.39	.568	1.249	0.582 ± 2.683
PLT (10^9^/L)	−0.008	0.006	.169	0.992	0.981 ± 1.003

APTT = activated partial thromboplastin time, CI = confidence interval, FIB = fibrinogen, OR = odds ratio, PLT = platelet, PT = prothrombin time, SE = standard error.

* *P* < .05.

**Figure 1.**
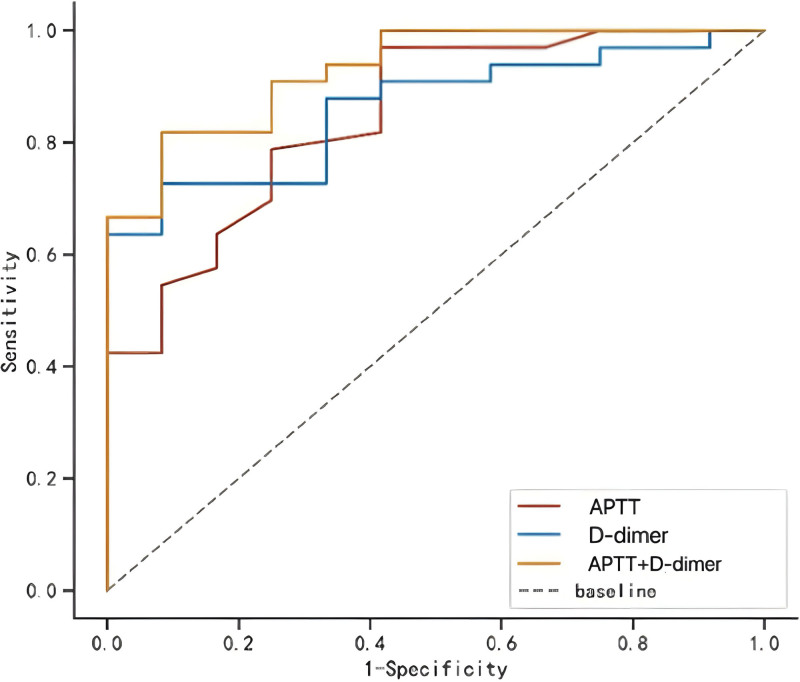
ROC curve model of APTT and D-dimer in HS. The optimal threshold of D-dimer is 568 µg/L, and the optimal threshold of APTT is 30.5 s. APTT + D-dimer: combining 2 indicators in heatstroke. APTT = activated partial thromboplastin time, HS = heatstroke, ROC = receiver operating characteristic.

The ROC curves are shown in Figure 1 and Table 4. Both D-dimer and APTT have high sensitivity and specificity in identifying HS, with areas under the curve (AUC) of 0.861 and 0.846, respectively. The combination of D-dimer and APTT showed higher specificity with an AUC of 0.929.

**Table 4:** The sensitivity and specificity of APTT and D-dimer in heatstroke were calculated by the receiver operating characteristic curve.

	Cutoff*	Sensitivity (%)	Specifity (%)	AUC	*P* value	95% CI
APTT (s)	30.5	97.1	58.3	0.846	.000	0.72–0.972
D-dimer (µg/L)	568	72.7	91.7	0.861	.000	0.754–0.968
APTT + D-dimer†	0.929	81.8	91.7	0.929	.000	0.854–1.000

APTT = activated partial thromboplastin time, AUC = area under the curve, CI = confidence interval.

* Threshold AUC.

† Combining 2 indicators in heatstroke.

### 3.3. Comparison of case fatality rate

In this study, as of the follow-up date, the overall 28-day fatality rate of the HS group was 66.7%, which was significantly higher than that of the control group (8.30%), but there was no significant difference in mortality between CHS and EHS components.

## 4. Discussion

Protective measures should be taken to prevent the occurrence of HS in different populations. Heat-related illnesses can be divided into heat cramps, heat exhaustion, and HS, of which HS is the most serious type. CHS is predominant in the elderly or patients with basic diseases. Young people who are engaged in manual labor under high temperature conditions are more likely to suffer from EHS.^[4]^ Our results of the general situation of patients also conformed to this epidemiological feature.

The survival condition in different types of heat-related illnesses was also different. The overall 28-day mortality rate of the HS group was 66.7%, which was higher than that of most studies in China.^[5–7]^ Age may be an important factor in the high mortality rate in our study. The average age of patients enrolled in this study was 68.27 ± 12.06 years, with 30 patients aged over 65 years (66.67%). The prognosis of severe HS in elderly patients was even worse.^[8,9]^ A previous study found that the mortality rate of CHS and EHS was 63.2% and 21.5%, respectively.^[10]^ However, our study found that the mortality rates of the CHS and EHS groups were 70.0% and 61.5%, respectively, with no significant difference between the 2 groups. There may be 3 reasons for this result: first, there was no significant difference in the overall age distribution, and the high mortality rate caused by advanced age masked the difference in mortality rates of the 2 groups. Second, it may be due to the insufficient sample size. Finally, regional development and medical conditions may also be important reasons for the high mortality rates.

The main pathophysiological mechanism of body damage caused by hyperthermia is a very complex process, and the injury of endothelial cells and the formation of diffuse microthrombus are prominent features of thermal radiation disease.^[11]^ Clinical observation has found that patients with HS often show thrombocytopenia, PT prolongation, and D-dimer increase. In animal experimental models, it has been found that high temperature can increase the levels of PT, APTT, D-dimer, and interleukin-6 in primates and thrombocytopenia.^[12]^ In this study, it was found that platelets were reduced to varying degrees in the typical febrile ejection groups, especially in the CHS group. Studies have shown that thrombocytopenia is also an important feature of febrile ejection, and early changes in platelets may be related to acute liver injury in febrile ejection.^[13]^ The inability to continuously and dynamically compare the relationship between changes in coagulation indexes and liver damage is one of the shortcomings of this study.

After correlation analysis of retrospectively collected data, it is found that abnormal results of coagulation indexes are not directly related to liver damage. Considering the existing studies on the pathogenesis of heat attack disease, abnormal coagulation may be related to extensive vascular endothelial damage. Systemic inflammatory response was more likely to be associated. The D-dimer in the ESH group was significantly higher than that in the other 2 groups, which was consistent with previous domestic research results,^[14]^ especially the high EHS sensitivity.^[15]^ However, this study found that APTT was generally prolonged upon admission, which may be related to the early depletion of coagulation factors.

Although the difference was not significant among the different types of thermal radiation disease, we further grouped all HS patients and compared them with the control group and found that APTT in the HS group was significantly higher than that in the control group, which was also consistent with the changes in coagulation in the pathogenesis of HS. That is, microthrombus formation due to vascular endothelial injury.^[16]^ The ROC curve indicated that these 2 indexes were of high value in helping identifying HS with AUCs of 0.861 and 0.846, respectively, which had certain reference value for clinical judgment of the degree of HS. At the same time, the abnormality of the above indicators means a higher DIC score. According to reports, the incidence of DIC in HS is as high as 48%, and recent studies have found that the occurrence of DIC is highly correlated with the prognosis of HS.^[1,17]^

However, the management of coagulation system in patients with HS is still a difficult problem. Studies have shown that lowering the core temperature may be effective in inhibiting fibrinolysis but not in inhibiting the sustained activation of coagulation, which is similar to sepsis.^[18]^ Due to the large consumption of coagulation factors in the early stage of DIC, although APTT is prolonged and D-dimer is elevated, anticoagulants may still be needed to inhibit the continued activation of the coagulation pathway. At the same time, it may be necessary to supplement large amounts of coagulation factors such as fresh frozen plasma to balance the fibrinolytic activation and the level of plasminogen activator inhibitor-1.^[19]^ In coagulation abnormalities caused by thermal injury, the need for anticoagulant or procoagulant therapy depends on the dynamic level between coagulation and fibrinolytic systems, as well as the stage of DIC.

Recent studies suggest that anticoagulation therapy may be beneficial to improve the survival time of patients with febrile radiation.^[20]^ Therefore, judging the degree of HS and the risk of DIC by early coagulation conditions can provide references for anticoagulation treatment strategies. Our study shows that APTT and D-dimer can be used as markers of HS to help judge the severity of the disease in the early stage of the disease, but our sample size is small, so a larger sample size is still needed to calculate the accurate range of APTT and D-dimer. Although we used different statistical methods, we still need to expand the sample size to confirm this study. In addition, the follow-up period of this study should be further extended to analyze the effect of changes in coagulation parameters on long-term prognosis.

## 5. Conclusion

The mortality rate of HS is extremely high, and elderly patients may have a worse prognosis. Prevention may be the main means to reduce the morbidity. Different types of HS have different clinical characteristics. Exertional HS mainly occurs in young men, and classical HS mainly occurs in elderly patients. APTT and D-dimer are related to the severity of HS to a certain extent and can be used as the makers of HS.

## Author contributions

**Methodology:** Xu Li.

**Project administration:** Xu Li.

**Resources:** Xu Li.

**Software:** Xu Li, Yuanjie Wang.

**Conceptualization:** Yuanjie Wang.

**Supervision:** Yuanjie Wang.

**Writing – original draft:** Yuanjie Wang.

## References

[1] Hifumi T Kondo Y Shimazaki J. Prognostic significance of disseminated intravascular coagulation in patients with heatstroke in a nationwide registry. J Crit Care. 2018;44:306–11. 29253838 10.1016/j.jcrc.2017.12.003

[2] Liu SY Song JC Mao HD Zhao JB Song Q; Expert Group of Heat Stroke Prevention and Treatment of the People’s Liberation Army, and People’s Liberation Army Professional Committee of Critical Care Medicine. Expert consensus on the diagnosis and treatment of heat stroke in China. Mil Med Res. 2020;7:1. 31928528 10.1186/s40779-019-0229-2 PMC6956553

[3] Gauer R Meyers BK. Heat-related illnesses. Am Fam Physician. 2019;99:482–9. 30990296

[4] Davis BC Tillman H Chung RT. Acute Liver Failure Study Group. Heatstroke leading to acute liver injury & failure: a case series from the Acute Liver Failure Study Group. Liver Int. 2017;37:509–13. 28128878 10.1111/liv.13373 PMC5516922

[5] Pan MZ Xu HH Dong CY Zhou XD Zhang JH Qian HL. [Analysis on influencing factors of deaths from severe heatstroke in Shanghai, 2013-2017]. Zhonghua Yu Fang Yi Xue Za Zhi. 2019;53:93–6. 30605969 10.3760/cma.j.issn.0253-9624.2019.01.013

[6] Wu M Wang C Liu Z Liu Z. Sequential organ failure assessment score for prediction of mortality of patients with rhabdomyolysis following exertional heatstroke: a longitudinal cohort study in Southern China. Front Med (Lausanne). 2021;8:724319. 34708052 10.3389/fmed.2021.724319 PMC8542709

[7] Liu Q Li C. [Predictive value of myoglobin and D-dimer on severe heatstroke: a clinical analysis of 38 patients with severe heatstroke]. Zhonghua Yu Fang Yi Xue Za Zhi. 2019;31:594–7. 10.3760/cma.j.issn.2095-4352.2019.05.014 31198146

[8] Epstein Y Yanovich R. Heatstroke. N Engl J Med. 2019;380:2449–59. 31216400 10.1056/NEJMra1810762

[9] Vaidyanathan A Malilay J Schramm P Saha S. Heat-related deaths - United States, 2004-2018. MMWR Morb Mortal Wkly Rep. 2020;69:729–34. 32555133 10.15585/mmwr.mm6924a1 PMC7302478

[10] Bouchama A Abuyassin B Lehe C. Classic and exertional heatstroke. Nat Rev Dis Primers. 2022;8:8. 35115565 10.1038/s41572-021-00334-6

[11] Iba T Connors JM Levi M Levy JH. Heatstroke-induced coagulopathy: biomarkers, mechanistic insights, and patient management. EClinicalMedicine. 2022;44:101276. 35128366 10.1016/j.eclinm.2022.101276 PMC8792067

[12] Bouchama A Roberts G Al MF. Inflammatory, hemostatic, and clinical changes in a baboon experimental model for heatstroke. J Appl Physiol (1985). 2005;98:697–705. 15475604 10.1152/japplphysiol.00461.2004

[13] Liu A Pu Z Chu L Ding H Zhou Y. [Analysis of clinical characteristics and risk factors of early heat stroke-related acute liver injury]. Zhonghua Wei Zhong Bing Ji Jiu Yi Xue. 2023;35:724–9. 37545450 10.3760/cma.j.cn121430-20230301-00128

[14] Wang L Jia H Shen Y. Diagnostic significance of combined calcitoninogen, platelet, and D-dimer assay in severe heatstroke: with clinical data analysis of 70 patients with severe heatstroke. Ther Hypothermia Temp Manag. 2023;13:29–37. 36067330 10.1089/ther.2022.0011

[15] Wu M Wang C Liu Z. Clinical characteristics and risk factors associated with acute kidney injury inpatient with exertional heatstroke: an over 10-year intensive care survey. Front Med (Lausanne). 2021;8:678434. 34095181 10.3389/fmed.2021.678434 PMC8170299

[16] Bouchama A Knochel JP. Heatstroke. N Engl J Med. 2002;346:1978–88. 12075060 10.1056/NEJMra011089

[17] Shimazaki J Hifumi T Shimizu K. Clinical characteristics, prognostic factors, and outcomes of heat-related illness (Heatstroke Study 2017-2018). Acute Med Surg. 2020;7:e516. 32551124 10.1002/ams2.516 PMC7298290

[18] Bouchama A Bridey F Hammami MM. Activation of coagulation and fibrinolysis in heatstroke. Thromb Haemost. 1996;76:909–15. 8972010

[19] Iba T Helms J Levi M Levy JH. Inflammation, coagulation, and cellular injury in heat-induced shock. Inflamm Res. 2023;72:463–73. 36609608 10.1007/s00011-022-01687-8

[20] Walter E Gibson OR. The efficacy of steroids in reducing morbidity and mortality from extreme hyperthermia and heatstroke-a systematic review. Pharmacol Res Perspect. 2020;8:e626. 10.1002/prp2.626 PMC7360483 32666709

